# Linkages between lifestyle practices, dietary habits, and premenstrual syndrome among college students

**DOI:** 10.1371/journal.pgph.0007266

**Published:** 2026-09-15

**Authors:** Mashael Huwaikem

**Affiliations:** Department of Clinical Nutrition, King Faisal University, Al Hofuf, Saudi Arabia; China Medical University College of Public Health, CHINA

## Abstract

Premenstrual syndrome (PMS) is a common condition characterized by physical, emotional, and behavioral symptoms that occur during the luteal phase of the menstrual cycle. Emerging evidence suggests that dietary and lifestyle factors may be associated with the occurrence and severity of PMS. A cross-sectional study was conducted among 502 female undergraduate students recruited through a convenience sampling approach at King Faisal University, Saudi Arabia. Data were collected using a self-administered questionnaire, the Shortened Premenstrual Assessment Form (SPAF), and a Food Frequency Questionnaire (FFQ). Associations between self-reported PMS symptoms, dietary habits, and lifestyle factors were examined using correlation analyses, group comparisons, and multivariable logistic regression. The mean BMI of the participants was 22.5 ± 5.1 kg/m^2^, and 62.0% met the predefined SPAF threshold (score ≥27) for clinically significant self-reported PMS symptoms. Regular physical activity was significantly more common among participants below the SPAF threshold. BMI was weakly but significantly associated with PMS symptom severity (r = 0.111, p = 0.013). Multivariable logistic regression analysis identified smoking, self-perceived dysmenorrhea, and the consumption of burgers, hard cheese, oranges, potato chips, coffee, and tea as factors associated with higher odds of clinically significant self-reported PMS symptoms. No significant differences were observed between participants below and above the SPAF threshold regarding age, overall daily caffeine intake, or daily water consumption. This study identified modest associations between selected dietary habits, lifestyle factors, and clinically significant self-reported PMS symptoms among female university students. Regular exercise was associated with lower odds of clinically significant self-reported PMS symptoms, whereas smoking and the consumption of several food items were associated with higher odds. Given the cross-sectional nature of the study, causal relationships cannot be established. Further longitudinal and intervention studies are needed to clarify the direction and magnitude of these associations.

## Introduction

Menstruation is a normal physiological process that occurs throughout the reproductive years and is regulated by cyclical hormonal changes. Premenstrual syndrome (PMS) is a common condition characterized by a constellation of physical, emotional, cognitive, and behavioral symptoms that occur during the late luteal phase of the menstrual cycle and typically resolve shortly after the onset of menstruation. Fluctuations in ovarian hormones, particularly estrogen and progesterone, are believed to contribute to the development of PMS symptoms [1]. Although symptom severity varies considerably among individuals, PMS can substantially affect daily functioning and quality of life.

The global prevalence of PMS has been estimated at approximately 48% among women of reproductive age [2]. However, prevalence rates vary considerably across populations and geographic regions. Studies conducted in Arab countries have reported relatively high prevalence rates, including 72.9% among Jordanian women and 77.7% among Egyptian women [3]. PMS is associated with a broad spectrum of symptoms, including irritability, fatigue, mood fluctuations, anxiety, breast tenderness, abdominal pain, headaches, and gastrointestinal disturbances [4,5]. Emerging evidence suggests that dietary composition and eating behaviors may influence both the occurrence and severity of PMS symptoms [5].

Among adolescents and young adults, PMS may adversely affect academic performance, social functioning, psychological well-being, and overall quality of life. Previous studies have reported associations between PMS and reduced academic achievement, impaired work-related quality of life, increased healthcare utilization, and decreased athletic performance [6–8]. Furthermore, PMS has been linked to emotional and cognitive disturbances that may interfere with everyday activities and interpersonal relationships [9].

The etiology of PMS is multifactorial and involves interactions among biological, genetic, environmental, and lifestyle-related factors [10]. Among these factors, dietary habits have received increasing attention because they represent potentially modifiable behaviors. Previous studies have suggested that dietary patterns rich in refined carbohydrates, saturated fats, and highly processed foods may be associated with a higher prevalence of PMS, whereas healthier dietary patterns may be associated with fewer symptoms [11,12]. In addition, cultural and social environments may influence dietary practices, symptom perception, and health-seeking behaviors, potentially contributing to variations in PMS prevalence and symptom expression across populations [13].

Despite growing interest in the relationship between diet and PMS, findings remain inconsistent. Helmy et al. [14] reported no significant associations between most dietary factors and PMS severity, whereas [15] found that women with PMS consumed greater amounts of fats and simple carbohydrates and lower amounts of protein. Moreover, the increasing availability and accessibility of highly processed and energy-dense foods have altered dietary patterns worldwide and may contribute to adverse health outcomes [16].

Recent studies from Saudi Arabia and other Gulf countries have confirmed that PMS remains a significant public health concern among university students. A recent cross-sectional study conducted among female students at King Khalid University in Saudi Arabia demonstrated that PMS symptom severity was significantly associated with body mass index, menstrual characteristics, and selected sociodemographic factors, emphasizing the continuing burden of PMS among young Saudi women [17]. Similarly, studies conducted in the Gulf region have identified associations between PMS and modifiable lifestyle factors, including unhealthy dietary habits, smoking, physical inactivity, and anthropometric characteristics [15,18]. Collectively, these findings indicate that both biological and lifestyle factors contribute to PMS; however, the reported associations remain inconsistent across populations because of differences in study design, assessment tools, and the range of variables examined.

Although previous Saudi and Gulf studies have provided valuable information regarding PMS prevalence and selected associated factors, most have primarily focused on symptom prevalence, menstrual characteristics, or a limited number of lifestyle variables. Few studies have comprehensively examined habitual dietary intake, individual food and beverage consumption, physical activity, smoking status, body mass index, hydration practices, and menstrual characteristics simultaneously within a single multivariable analytical framework. Furthermore, unlike previous Saudi studies that primarily examined prevalence or selected demographic factors, the present study simultaneously evaluated multiple modifiable dietary and lifestyle factors within a single multivariable analytical framework, while providing evidence from the Eastern Province of Saudi Arabia, where published data remain limited.

Therefore, the present study extends the existing literature by simultaneously evaluating dietary habits, lifestyle practices, body mass index, hydration practices, and menstrual characteristics using two validated instruments, the Shortened Premenstrual Assessment Form (SPAF) and the Food Frequency Questionnaire (FFQ), in a relatively large cohort of female undergraduate students from King Faisal University. Unlike previous regional studies that mainly estimated prevalence or evaluated isolated risk factors, the present study provides a comprehensive assessment of multiple modifiable dietary and lifestyle factors associated with clinically significant self-reported PMS symptoms. These findings provide region-specific evidence that may support the development of targeted nutritional and lifestyle interventions for female university students in Saudi Arabia.

The Eastern Province offers a valuable target for studying health and menstrual outcomes in young Saudi females. Data from the 2022 Saudi Census show that the region has a notably young population, and it is also marked by considerable urbanization and socioeconomic variety, with major cities like Dammam, Al-Ahsa, Al-Khobar, Qatif, and Jubail [19]. Further, these features mean the area contains a considerable group of young adults who may be experiencing changing diets and lifestyles. Regarding menstrual health specifically, earlier research by Alali Z. [20] in this population found that among 500 participants, 32.2% experienced premenstrual syndrome and 22.6% experienced premenstrual dysphoric disorder, with these conditions leading to reduced quality of life across physical, psychological, social, and environmental areas. More recently investigations in this region indicates a considerable burden of menstrual problems, including dysmenorrhea, along with links to irregular cycles, heavy flow, higher BMI, and limited functionality. Taking these facts in account, the evidences establish menstrual health as a significant concern for young women in the Eastern Province and underscores the value of investigating modifiable factors—particularly diet and lifestyle—in relation to PMS.

Therefore, the present study aimed to examine the associations between dietary habits and lifestyle factors including physical activity, smoking status, body mass index (BMI), and hydration practices and clinically significant self-reported PMS symptoms among female university students at King Faisal University. We hypothesized that unhealthy dietary habits and adverse lifestyle behaviors would be associated with higher odds of meeting the predefined SPAF threshold.

## Materials and methods

### Ethics statement

A clear assurance regarding the secure and confidential handling of all information collected throughout the study was provided to each woman taking part. To protect their privacy, names and home addresses were intentionally left unrecorded during data acquisition. The investigation received ethical authorization from the Research Ethics Committee of King Faisal University, recorded under Reference No. KFU-REC-2024-JAN-ETHICS1965.

There were no incentives offered whatsoever, either in financial or non-financial form. Prior to signing the consent paperwork, every woman was given a complete oral overview of what the study was aiming to achieve and what it would cover.

#### Design, setting and participants.

This cross-sectional study was conducted among female undergraduate students enrolled at King Faisal University (KFU), Saudi Arabia. The required sample size was determined through a priori power analysis, and a total of 502 eligible female undergraduate students were recruited using a convenience sampling approach.

Eligible participants were female students aged 18–30 years, enrolled at KFU during the study period, reporting regular menstrual cycles, and willing to provide informed consent. Participants were excluded if they were pregnant, had a history of chronic medical conditions (e.g., diabetes mellitus or uterine fibroids), had a diagnosed psychiatric disorder, or were outside the specified age range. These eligibility criteria were applied to ensure a relatively homogeneous study population and to minimize potential confounding factors that might influence premenstrual symptoms.

Demographic and health-related information collected from participants included age, body mass index (BMI), smoking status, physical activity, age at menarche, diagnosis of dysmenorrhea, self-perceived dysmenorrhea, breakfast consumption, caffeine intake, water intake, and history of weight-loss dieting.

Regular exercise was defined as participation in moderate-to-vigorous physical activity at least three times per week for a minimum of 30 minutes per session. Sweet and salty food preferences were assessed using self-reported preference questions and reflected participants’ usual liking for these foods rather than actual dietary intake.

Dietary intake was assessed using a Food Frequency Questionnaire (FFQ) that included a wide range of food items from major food groups, including fruits, vegetables, dairy products, beverages, snacks, and fast foods. Participants reported their usual food consumption frequency during the previous three months. The FFQ was designed to evaluate habitual dietary intake and was not restricted to a specific phase of the menstrual cycle. For clarity, Table 5 presents the dietary variables retained in the final multivariable model, including statistically significant variables and olive oil consumption, which approached but did not reach statistical significance.

### Study’s tools

In addition to collecting demographic and health-related information, the study utilized two validated instruments to assess premenstrual symptoms and dietary habits. Demographic and health-related characteristics included age, body mass index (BMI), smoking status, physical activity, age at menarche, physician-diagnosed dysmenorrhea, self-perceived dysmenorrhea, daily water intake, caffeine consumption, breakfast consumption habits, and history of weight-loss dieting. These variables were included because of their potential association with premenstrual syndrome and were subsequently considered in the statistical analyses.

### Tool I: Shortened premenstrual assessment form (SPAF)

Premenstrual symptom severity was assessed using the Shortened Premenstrual Assessment Form (SPAF) developed by Allen and McBride [21]. The instrument consists of 10 items scored on a six-point Likert scale, yielding a total score ranging from 10 to 60, with higher scores indicating greater symptom severity. Consistent with previous studies, a total SPAF score of ≥27 was used to identify participants reporting clinically significant premenstrual symptoms. The SPAF is a validated screening instrument for assessing self-reported PMS symptom severity and was used to classify symptom burden rather than to establish a clinical diagnosis of PMS or Premenstrual Dysphoric Disorder (PMDD). [22].

### Tool II: Food frequency questionnaire (FFQ)

Dietary intake was assessed using a validated Food Frequency Questionnaire (FFQ). Participants reported their usual frequency of food consumption during the previous three months. The FFQ assessed habitual dietary intake across major food groups and was not restricted to a specific menstrual cycle phase. The instrument has been widely used and validated for dietary assessment [23,24].

### Data collection

Following an extensive review of the literature, the study instruments were developed, and their reliability was assessed using Cronbach’s alpha coefficients. The internal consistency was satisfactory for both the Shortened Premenstrual Assessment Form (SPAF; α = 0.904) and the Food Frequency Questionnaire (FFQ; α = 0.856). Prior to the main study, a pilot test involving 30 female undergraduate students was conducted to evaluate the clarity, feasibility, and relevance of the study instruments.

Based on the activities schedule provided by the Deanship of Student Affairs, eligible female students were approached and invited to participate. Before completing the questionnaire, all participants were screened for eligibility using a structured screening form based on the predefined inclusion and exclusion criteria. Eligibility was confirmed by verifying participants’ age, undergraduate enrollment status, regular menstrual cycles, pregnancy status, and self-reported history of chronic medical or psychiatric conditions. Only eligible students who provided written informed consent were invited to complete the study questionnaire.

Data collection was conducted between February and April 2024. Data were collected using a self-administered paper-based questionnaire completed independently by the participants in the presence of the researcher, who was available only to clarify procedural questions and did not influence participants’ responses. No face-to-face interviews were conducted. Participants self-reported their height and body weight in the questionnaire, and body mass index (BMI) was subsequently calculated as weight in kilograms divided by the square of height in meters (kg/m^2^). Personal identifiers were not collected, and all data were de-identified prior to analysis to ensure confidentiality. As this was a cross-sectional study, questionnaire administration was not scheduled according to a specific menstrual cycle phase. Participants were instructed to report their usual premenstrual symptoms and habitual dietary practices experienced across recent menstrual cycles rather than symptoms related to a single menstrual cycle. The entire data collection process was completed over a three-month period.

### Data analysis

Statistical analyses were performed using IBM SPSS Statistics version 26. Descriptive statistics were used to summarize participant characteristics. Categorical variables were presented as frequencies and percentages, whereas continuous variables were reported as means ± standard deviations (SD) or medians and interquartile ranges (IQR), as appropriate.

Associations between categorical variables were assessed using the Chi-square test. Group comparisons were performed using the independent-samples t-test or Mann–Whitney U test, depending on data distribution. Spearman’s rank correlation coefficients were calculated for ordinal and continuous variables, whereas point-biserial correlation coefficients were used for dichotomous variables.

Variables demonstrating a significance level of *p* < 0.10 in the univariate analyses were entered into the multivariable logistic regression model. Age was additionally retained a priori because of its potential confounding role, irrespective of its univariate statistical significance. The final model therefore included age, BMI, regular exercise, smoking status, self-perceived dysmenorrhea, weight-loss dieting, and the dietary variables identified from the Food Frequency Questionnaire (burger, hard cheese, orange, potato chip, coffee, tea, and olive oil consumption frequencies). Variables that were not collected in this study, including psychological stress, sleep quality, total caloric intake, and other potential confounders, were not available for adjustment.

Results are presented as adjusted odds ratios (AORs) with 95% confidence intervals (95% CI). Statistical significance was set at p < 0.05. Missing data were handled using listwise deletion. Given the exploratory nature of the study, no formal correction for multiple comparisons was applied; therefore, findings should be interpreted cautiously.

## Results

A summary of the demographic and health-related characteristics of the 502 participants is presented in Table 1. More than half of the participants (55.4%) were aged 18–20 years. The mean body mass index (BMI) was 22.5 ± 5.1 kg/m^2^, indicating that, on average, participants were within the normal weight range. Regarding lifestyle characteristics, only 20.3% of participants reported engaging in regular exercise, whereas 79.7% reported no regular physical activity. Smoking prevalence was low, with only 4.4% of participants reporting current smoking.

**Table 1:** Demographic and Clinical Characteristics of the Participants (N = 502).

Characteristics	Frequency (n = 502)	Percentage (%)
**Age**		
18–20 years	278	55.4
21–22 years	114	22.7
23–25 years	62	12.4
26–28 years	48	9.6
**BMI (kg/m²)**	Mean ± SD	22.5 ± 5.1
**Regular exercise**		
Yes	102	20.3
No	400	79.7
**Smoking**		
Yes	22	4.4
No	480	95.6
**Age at menarche**		
<12 years	104	20.7
12–14 years	347	69.1
≥15 years	51	10.2
**Physician-diagnosed dysmenorrhea**		
Yes	16	3.2
No	486	96.8
**Self-perception of dysmenorrhea**		
Yes	55	11.0
No	447	89.0

Most participants (69.1%) reported menarche between 12 and 14 years of age, while 20.7% reported menarche before the age of 12 years and 10.2% reported menarche at 15 years of age or older. A clinical diagnosis of dysmenorrhea was reported by 3.2% of participants, whereas 11.0% self-reported experiencing dysmenorrhea symptoms.

Table 2 summarizes the dietary habits and food preferences of the participants. Regular breakfast consumption (≥5 days/week) was reported by 61.8% of participants. Most participants (80.7%) consumed one to two cups of caffeinated beverages per day. Regarding hydration practices, 34.1% reported consuming only one to two cups of water daily. Preferences for sweet foods (74.5%) and salty foods (70.5%) were commonly reported.

**Table 2:** Dietary Habits and Dietary Preferences among the Studied Participants.

	Frequency	Percentage
**Breakfast consumption (≥5 days/week)**		
*Yes*	310	(61.8%)
*No*	192	(38.2%)
**Weight loss diet in the last year**		
*Yes*	150	(29.9%)
*No*	352	(70.1%)
**Daily caffeine intake “200 ml per cup”**		
*1-2 cup /day*	405	(80.7%)
*3-5 cup / day*	79	(15.7%)
*>5 cup / day*	18	(3.6%)
**Daily water intake “200 ml per cup”**		
*1-2 cup /day*	171	(34.1%)
*3-5 cup /day*	210	(41.8%)
*6-8 cup /day*	99	(19.7%)
*9-12 cup /day*	22	(4.4%)
**Sweet food preferences**		
*Yes*	374	(74.5%)
*No*	128	(25.5%)
**Salty food preferences**		
*Yes*	354	(70.5%)
*No*	148	(29.5%)

Table 3 presents the associations between Premenstrual Assessment scores and participant characteristics. A weak but statistically significant positive association was observed between BMI and Premenstrual Assessment scores (r = 0.111, p = 0.013), indicating that participants with higher BMI tended to report greater symptom severity. Similarly, smoking status was positively associated with Premenstrual Assessment scores (r = 0.111, p = 0.013). In contrast, regular exercise demonstrated a weak negative association with Premenstrual Assessment scores (r = −0.102, p = 0.022), suggesting lower symptom scores among participants who engaged in regular physical activity. Self-perceived dysmenorrhea was also positively associated with Premenstrual Assessment scores (r = 0.134, p = 0.003).

**Table 3:** Associations Between Premenstrual Assessment Scores and Participant Characteristics.

Variable	Correlation Coefficient (r)	P-value
Age	0.053	0.236
BMI	0.111	0.013*
Regular exercise	-0.102	0.022*
Smoking	0.111	0.013*
Breakfast consumption (≥5 days/week)	0.001	0.996
Weight-loss diet during the previous year	0.077	0.085
Daily caffeine intake (cups/day)	0.032	0.477
Daily water intake (cups/day)	0.028	0.534
Age at menarche	-0.031	0.484
Physician-diagnosed dysmenorrhea	-0.016	0.717
Self-perceived dysmenorrhea	0.134	0.003*
Sweet food preference	0.006	0.898
Salty food preference	-0.005	0.919

*Statistically significant at p < 0.05.

**Note:** Variables entered into the multivariable logistic regression model included age (retained a priori because of its potential confounding role), BMI, regular exercise, smoking status, self-perceived dysmenorrhea, weight-loss dieting, and the following dietary variables: burger, hard cheese, orange, potato chip, coffee, tea, and olive oil consumption frequencies. Variables not collected in this study, including psychological stress, sleep quality, and total caloric intake, were not available for adjustment.

No statistically significant associations were observed between Premenstrual Assessment scores and age, breakfast consumption, weight-loss dieting, daily caffeine intake, daily water intake, age at menarche, physician-diagnosed dysmenorrhea, sweet food preference, or salty food preference (all p > 0.05). Overall, the observed associations were weak in magnitude (|r| < 0.20), indicating small effect sizes despite statistical significance.

Based on the predefined SPAF cutoff score of ≥27, 311 participants (62.0%) met the threshold for clinically significant self-reported PMS symptoms, whereas 191 participants (38.0%) scored below this threshold.

Table 4 compares demographic, lifestyle, and dietary characteristics between participants below and above the predefined SPAF threshold. Regular exercise was significantly more common among participants below the SPAF threshold than among those who met the threshold for clinically significant self-reported PMS symptoms (25.1% vs. 17.4%, p = 0.036). Conversely, smoking was more prevalent among participants who met the SPAF threshold than among those below the threshold (6.4% vs. 1.0%, p = 0.004). Self-perceived dysmenorrhea was also significantly more common among participants who met the SPAF threshold than among those below the threshold (13.8% vs. 6.3%, p = 0.009). No statistically significant differences were observed between the two groups with respect to age, BMI, breakfast consumption, weight-loss dieting, overall daily caffeine intake, daily water intake, age at menarche, physician-diagnosed dysmenorrhea, sweet food preference, or salty food preference (all p > 0.05). Overall, regular exercise, smoking status, and self-perceived dysmenorrhea were significantly associated with meeting the predefined SPAF threshold for clinically significant self-reported PMS symptoms.

**Table 4:** Comparison of Demographic, Lifestyle, and Dietary Characteristics According to the SPAF Threshold for Clinically Significant Self-Reported PMS Symptoms.

Variable	Below SPAF threshold (n = 191)	Met SPAF threshold (n = 311)	P-value
**Age**			0.291
18–20 years	115 (60.2%)	163 (52.4%)	
21–22 years	37 (19.4%)	77 (24.8%)	
23–25 years	20 (10.5%)	42 (13.5%)	
26–28 years	19 (9.9%)	29 (9.3%)	
**BMI (kg/m²)**	22.1 ± 5.5	22.7 ± 4.9	0.220
**Regular exercise**			0.036*
Yes	48 (25.1%)	54 (17.4%)	
No	143 (74.9%)	257 (82.6%)	
**Smoking**			0.004*
Yes	2 (1.0%)	20 (6.4%)	
No	189 (99.0%)	291 (93.6%)	
**Breakfast consumption (≥5 days/week)**			0.455
Yes	114 (59.7%)	196 (63.0%)	
No	77 (40.3%)	115 (37.0%)	
**Weight-loss diet during the previous year**			0.105
Yes	49 (25.7%)	101 (32.5%)	
No	142 (74.3%)	210 (67.5%)	
**Daily caffeine intake (cups/day)**			0.368
1–2	157 (82.2%)	248 (79.7%)	
3–5	30 (15.7%)	49 (15.8%)	
>5	4 (2.1%)	14 (4.5%)	
**Daily water intake (cups/day)**			0.141
1–2	63 (33.0%)	108 (34.7%)	
3–5	90 (47.1%)	120 (38.6%)	
6–8	29 (15.2%)	70 (22.5%)	
9–12	9 (4.7%)	13 (4.2%)	
**Age at menarche**			0.841
<12 years	37 (19.4%)	67 (21.5%)	
12–14 years	134 (70.2%)	213 (68.5%)	
≥15 years	20 (10.5%)	31 (10.0%)	
**Physician-diagnosed dysmenorrhea**			0.569
Yes	5 (2.6%)	11 (3.5%)	
No	186 (97.4%)	300 (96.5%)	
**Self-perceived dysmenorrhea**			0.009*
Yes	12 (6.3%)	43 (13.8%)	
No	179 (93.7%)	268 (86.2%)	
**Sweet food preference**			0.435
Yes	146 (76.4%)	228 (73.3%)	
No	45 (23.6%)	83 (26.7%)	
**Salty food preference**			0.950
Yes	135 (70.7%)	219 (70.4%)	
No	56 (29.3%)	92 (29.6%)	

*Statistically significant at *p* < 0.05.

Multivariable logistic regression identified several factors independently associated with clinically significant self-reported PMS symptoms (Table 5). Regular exercise was associated with lower odds of meeting the predefined SPAF threshold (AOR = 0.626, 95% CI: 0.030–0.971, p = 0.037). In contrast, smoking (AOR = 6.495, 95% CI: 1.501–28.108, p = 0.012) and self-perceived dysmenorrhea (AOR = 2.393, 95% CI: 1.228–4.664, p = 0.010) were associated with higher odds of meeting the threshold.

**Table 5:** Multivariable Logistic Regression Analysis of Factors Associated with Clinically Significant Self-Reported PMS Symptoms Based on the SPAF Cutoff Score.

Variable	Adjusted odds ratio (AOR)	95% confidence interval	*P*-value
Regular exercise (yes vs. no)	0.626	0.030–0.971	0.037*
Smoking (yes vs. no)	6.495	1.501–28.108	0.012*
Self-perceived dysmenorrhea (yes vs. no)	2.393	1.228–4.664	0.010*
Burger consumption frequency	1.166	1.029–1.320	0.016*
Hard cheese consumption frequency	1.157	1.037–1.292	0.009*
Orange consumption frequency	1.144	1.026–1.278	0.015*
Potato chip consumption frequency	1.139	1.022–1.268	0.019*
Coffee consumption frequency	1.106	1.012–1.208	0.026*
Tea consumption frequency	1.118	1.019–1.228	0.019*
Olive oil consumption frequency	1.098	0.994–1.213	0.065

*Statistically significant at *p* < 0.05.

**Abbreviations:** AOR, adjusted odds ratio; CI, confidence interval; SPAF, Shortened Premenstrual Assessment Form.

**Note:** Variables entered into the multivariable logistic regression model included age (retained a priori because of its potential confounding role), BMI, regular exercise, smoking status, self-perceived dysmenorrhea, weight-loss dieting, and the following dietary variables: burger, hard cheese, orange, potato chip, coffee, tea, and olive oil consumption frequencies. Variables not collected in this study, including psychological stress, sleep quality, and total caloric intake, were not available for adjustment. Adjusted odds ratios greater than 1 indicate higher odds of meeting the predefined SPAF threshold (score ≥27) for clinically significant self-reported PMS symptoms, whereas adjusted odds ratios less than 1 indicate lower odds of meeting this threshold.

Among the dietary variables, higher consumption of burgers (AOR = 1.166, 95% CI: 1.029–1.320, p = 0.016), hard cheese (AOR = 1.157, 95% CI: 1.037–1.292, p = 0.009), oranges (AOR = 1.144, 95% CI: 1.026–1.278, p = 0.015), potato chips (AOR = 1.139, 95% CI: 1.022–1.268, p = 0.019), coffee (AOR = 1.106, 95% CI: 1.012–1.208, p = 0.026), and tea (AOR = 1.118, 95% CI: 1.019–1.228, p = 0.019) were independently associated with higher odds of clinically significant self-reported PMS symptoms. Olive oil consumption was not significantly associated with meeting the predefined SPAF threshold (AOR = 1.098, 95% CI: 0.994–1.213, p = 0.065). Overall, the associations observed for the individual dietary variables were modest, whereas the association with smoking was larger but had a wide confidence interval and should therefore be interpreted cautiously. Although overall daily caffeine intake, categorized by the number of caffeinated beverages consumed per day, was not significantly associated with meeting the predefined SPAF threshold in the univariate analysis, the multivariable logistic regression identified coffee and tea consumption frequencies as independent dietary factors associated with higher odds of clinically significant self-reported PMS symptoms. This difference likely reflects the distinct analytical approaches used. Daily caffeine intake represented a broad measure of total caffeinated beverage consumption, whereas the Food Frequency Questionnaire (FFQ) assessed the consumption frequency of individual food and beverage items. The multivariable model evaluated these individual dietary variables while simultaneously adjusting for other significant lifestyle and demographic factors, enabling beverage-specific associations to be identified.

## Discussion

Though PMS affects a substantial proportion of women during their reproductive years, its underlying etiology has not been fully elucidated. The present study was conducted to examine the associations between dietary habits, lifestyle practices, and clinically significant self-reported PMS symptoms among female undergraduate students while simultaneously considering menstrual and anthropometric characteristics.

Every participant in the present study reported experiencing one or more premenstrual symptoms, although symptom severity varied considerably. This finding is consistent with previous studies. Al Sabbah and Al Mutawa [18] likewise reported that all women in their cohort experienced at least one premenstrual symptom, whereas Jahrami et al. [25] reported a similarly high prevalence of symptoms (98.2%). The symptoms most frequently described in previous studies include fatigue, irritability, appetite changes, and musculoskeletal pain [26]. In addition, academic-related psychological stress may exacerbate premenstrual symptoms among university students, as reported among Lebanese female undergraduates [27].

The prevalence of clinically significant self-reported PMS symptoms observed in the present study (62.0%) was higher than the global prevalence estimate of approximately 48% reported in previous meta-analyses [2]. This difference may reflect variations in study populations, cultural contexts, assessment instruments, diagnostic criteria, and lifestyle characteristics. Similar prevalence rates have been reported among female university students in several Middle Eastern countries, suggesting that clinically significant premenstrual symptoms are particularly common within this demographic group [3,18].

Our findings are generally consistent with recent studies conducted in Saudi Arabia and other Gulf countries, which also reported a high prevalence of PMS and identified lifestyle and dietary factors as important correlates of symptom severity. However, unlike many previous regional studies that primarily focused on prevalence or a limited number of behavioral factors, the present study simultaneously evaluated dietary habits, lifestyle practices, BMI, and menstrual characteristics using validated SPAF and FFQ instruments within a multivariable analytical framework. Furthermore, this study provides evidence from the Eastern Province of Saudi Arabia, a region that remains underrepresented in the current literature.

Within the present analysis, regular exercise, smoking, and self-perceived dysmenorrhea emerged as factors independently associated with clinically significant self-reported PMS symptoms. Despite the relatively low prevalence of smoking among participants, smoking remained significantly associated with higher odds of clinically significant self-reported PMS symptoms, consistent with previous findings suggesting that tobacco use increases the likelihood and severity of premenstrual symptoms [28].

Although BMI showed a statistically significant positive correlation with self-reported PMS symptom severity, the magnitude of this association was small (r = 0.111). Therefore, the observed relationship should be interpreted cautiously because statistical significance does not necessarily indicate a clinically meaningful effect.

Dietary habits also emerged as important factors associated with clinically significant self-reported PMS symptoms. Higher consumption frequencies of coffee, tea, burgers, potato chips, hard cheese, and oranges were associated with higher odds of meeting the predefined SPAF threshold. Previous studies have similarly reported associations between caffeine consumption and PMS symptom severity [5,29,30], although Caan et al. [31] did not observe a significant association.

Overall caffeine intake, measured as the total number of caffeinated beverages consumed per day, was not significantly associated with meeting the predefined SPAF threshold in the univariate analysis. In contrast, coffee and tea consumption emerged as significant predictors in the multivariable model after adjustment for potential confounding variables. Because the FFQ evaluated the frequency of consumption for individual food and beverage items rather than total caffeine intake, these findings suggest that beverage-specific consumption patterns may better reflect dietary behaviors associated with clinically significant self-reported PMS symptoms than an aggregate measure of daily caffeine intake.

Our findings are also consistent with previous research demonstrating that Western dietary patterns and frequent fast-food consumption are associated with inflammatory biomarkers, whereas diets rich in antioxidants, vitamins, and phytochemicals may reduce systemic inflammation [11]. Because inflammatory mechanisms have been implicated in PMS pathophysiology, dietary habits may influence symptom severity through inflammatory pathways.

The dietary profile of the participants further illustrates the complexity of these associations. Approximately 61.8% reported consuming breakfast regularly, nearly 30% reported following a weight-loss diet during the previous year, and 80.7% reported consuming caffeinated beverages daily. Similar associations between PMS and disordered eating behaviors have been reported among adolescent and young adult women [32].

The results of this study can be compared well with recent findings from other parts of Saudi Arabia. According to Alshehri [33] assessing behavioral health including physical activity, eating pattern, nicotine use, and BMI—among 478 college students in the southern part of Saudi Arabia. The research concluded that 73.8% of students were physically active as per recommended levels, and 54.4% were presented with dietary behaviors as judged by the Food Frequency Scale. Mean BMI was 23.3 ± 6.3 kg/m^2^, with 47.5% had a healthy weight. Among female students, 28.6% were underweight, 46.5% were a healthy weight, and 23.3% were overweight or obese. As a contrast, current Eastern Province study participants had a lower mean BMI of 22.5 ± 5.1 kg/m^2^, and those without PMS were more likely to engage in regular physical activity. Furthermore, these results depict possible regional variation in lifestyle and nutritional patterns among Saudi university students. Direct comparisons should be judged with care because the research done in Southern Region included both male and female students, employed different instruments to conclude dietary and physical activity behaviors, and was designed to evaluate general behavioral health rather than PMS. Variety of food culture in different regions, urbanization patterns, socioeconomic conditions, and lifestyle behavior could help explain these differences in health behaviors and nutritional status. Further researches from different regions using similar, standardized measures are required to understand regional variation and its effect on menstrual health and PMS among Saudi female population in a better way.

Several biological mechanisms may explain the observed associations between diet and PMS. Diets rich in highly processed foods may promote systemic inflammation, oxidative stress, and altered neurotransmitter regulation, all of which have been implicated in PMS pathophysiology. High consumption of refined carbohydrates may contribute to glycemic fluctuations affecting mood and energy levels, whereas excessive sodium intake may exacerbate bloating and fluid retention. Conversely, nutrient-rich diets containing magnesium, calcium, vitamin B6, and antioxidants may support hormonal regulation and reduce symptom severity.

Because this study employed a cross-sectional design, the direction of the observed associations cannot be established. It is plausible that frequent consumption of highly processed foods, fast foods, and caffeinated beverages may contribute to the development or exacerbation of premenstrual symptoms through mechanisms such as inflammation, hormonal fluctuations, or altered neurotransmitter regulation. Conversely, women experiencing premenstrual symptoms may develop cravings for energy-dense, high-fat, high-sugar, or caffeinated foods as part of the physiological and psychological changes occurring during the premenstrual phase. Therefore, the observed associations are likely to be bidirectional, and longitudinal or prospective studies are needed to clarify the temporal relationship between dietary behaviors and PMS symptom development.

Taken together, these findings suggest that dietary habits may represent modifiable factors associated with clinically significant self-reported PMS symptoms. Individualized nutritional assessment and dietary counseling may help identify nutritional inadequacies and support targeted dietary interventions. Furthermore, diets rich in magnesium and B-group vitamins may contribute to reducing migraine symptoms associated with PMS [34].

### Limitations

Several limitations should be acknowledged. First, the cross-sectional design precludes causal inference. Second, dietary intake and premenstrual symptoms were self-reported and were therefore subject to recall bias. Participants may not have accurately recalled their habitual food consumption or the frequency and severity of premenstrual symptoms experienced across previous menstrual cycles. Consequently, recall bias may have influenced not only the assessment of dietary habits but also the overall reporting of PMS symptoms, which could have affected the observed associations.

In addition, nutritional assessment was limited to BMI as an anthropometric indicator. Other measures of nutritional status, such as waist circumference and biochemical markers (e.g., hemoglobin or serum ferritin to assess iron status), were not collected because of logistical and resource constraints. Future studies incorporating more comprehensive anthropometric and biochemical assessments may provide a more complete understanding of the relationship between nutritional status and PMS.

Finally, although the multivariable model adjusted for the variables collected in this study, residual confounding cannot be excluded. Potential confounders, including psychological stress, sleep quality, total caloric intake, socioeconomic status, and other lifestyle factors, were not measured and therefore could not be included in the adjusted analyses.

## Conclusion

This study identified modest associations between selected dietary habits, lifestyle factors, and clinically significant self-reported PMS symptoms among female university students. Regular exercise was associated with lower odds of clinically significant self-reported PMS symptoms, whereas smoking and the consumption of several food items were associated with higher odds. Because the study employed a cross-sectional design, causality and the temporal direction of the observed associations cannot be inferred. Future longitudinal and intervention studies are required to determine whether dietary habits influence PMS symptom severity, whether PMS symptoms alter dietary behaviors, or whether both mechanisms contribute to the observed relationships. These findings provide region-specific evidence from the Eastern Province of Saudi Arabia and may inform the development of targeted nutritional and lifestyle interventions aimed at reducing the burden of premenstrual symptoms among female university students.

## Abbreviations

PMS: premenstrual syndrome
SPAF: shortened premenstrual assessment form
FFQ: food frequency questionnaire
KFU: King Faisal University
BMI: body mass index
PMDD: premenstrual dysphoric disorder
OR: odds ratio
CI: confidence interval
SD: standard deviation
IQR: interquartile range
